# Top-50 cited articles on cysticercosis and neurocysticercosis

**DOI:** 10.1097/MD.0000000000037268

**Published:** 2024-03-01

**Authors:** Gregorio Gonzalez-Alcaide, Nestor Sosa, Fatima Valero-Samper, Isabel Belinchon-Romero, Jose-Manuel Ramos-Rincon

**Affiliations:** a Department of History of Science and Documentation, University of Valencia, Valencia, Spain; b Infectious Diseases Division, Internal Medicine Department, New Mexico University Health Sciences Center, Albuquerque, New Mexico, USA; c Internal Medicine Department, Dr. Balmis General University Hospital, Alicante Institute for Health and Biomedical Research (ISABIAL), Alicante, Spain; d Clinical Medicine Department, Miguel Hernández University, and Alicante Institute for Health and Biomedical Research (ISABIAL), Alicante, Spain.

**Keywords:** bibliometric, cysticercosis, neurocysticercosis, publications, research, *Taenia solium*

## Abstract

**Background:**

Identifying the most highly cited papers in a given field can help researchers and professionals understand the milestones and research areas that are generating the most impact. This study aimed to identify and describe the 50 most frequently cited manuscripts on cysticercosis and neurocysticercosis.

**Methods:**

We identified the 50 most cited papers (articles and reviews) on cysticercosis and neurocysticercosis from the MEDLINE database and indexed in Web of Science-Core Collection, analyzing their bibliographic and content characteristics.

**Results:**

The most cited documents comprised 29 (58%) original articles and 21 (42%) reviews, the bulk of which were narrative reviews (n = 17), with a negligible presence of other types of reviews with high-level scientific evidence. Six journals published 42% of the articles. In addition to the USA, Mexico and Peru were prominent countries of origin among leading researchers. The main research topics were the central nervous system and epilepsy on the one hand, and diagnostic and therapeutic approaches on the other.

**Conclusion:**

Our findings shed light on the dissemination of knowledge about cysticercosis and neurocysticercosis in recent decades, identifying the most highly cited contributions that have driven research in the field.

**Key points**

1. The review of the most important papers to help guide future research and practice in cysticercosis and neurocysticercosis.
2. This study identifies and reviews the top-cited articles on cysticercosis and neurocysticercosis
3. Our results show the relevance of neurology and general and internal medicine in the area; the research leadership of Peru and Mexico, together with the USA; and the keen research interest in the central nervous system & epilepsy, diagnostics, and therapeutic approaches in relation to cysticercosis and neurocysticercosis

## 1. Introduction

The growth in scientific knowledge on biomedicine has made it necessary to develop tools to manage and understand the body of evidence. Bibliometrics has become a well-established discipline for studying scientific activity and for describing the characteristics of different fields or areas of knowledge by quantifying the bibliographic characteristics of its literature.^[1]^ At present, identifying citation classics and top-cited papers are the main bibliometric methods for systematically assessing research performance. Reviewing the most impactful papers can help guide future research and practice. In that line, several medical specialties have ranked articles within their fields according to citation frequency.^[2–4]^

Cysticercosis and neurocysticercosis are endemic to large swaths of territory in Asia, sub-Saharan Africa, and Latin America and are associated with deprivation and poor sanitation and hygiene.^[5]^ Neurocysticercosis is responsible for 30% to 70% of epilepsies in some endemic countries and regions.^[5]^ The diagnosis and antiparasitic treatment to reduce the rate of seizures due to neurocysticercosisis a relevant topic on research due to the public health problem.^[5]^ This important public health challenge has thus attracted increasing research in the past several decades, but few studies have analyzed the scientific production on the diseases.^[6,7]^ Reviewing the most impactful papers can help guide future research and practice so no studies have analyzed the top-cited documents in the literature. This information can help optimize the allocation of resources, reorient research support, rationalize research organizations, restrict research in particular fields, and augment research productivity.^[2–4]^ The review of the most important papers to help guide future research and practice in cysticercosis and neurocysticercosis.

This study identifies and reviews the top-cited articles on cysticercosis and neurocysticercosis. We describe and analyze their characteristics with an eye to determining the factors that have made them the most relevant papers for other researchers working in the field. This information can help researchers and professionals understand the milestones and the research areas that are generating the most impact.

## 2. Material and methods

Bibliographic searches were performed in the MEDLINE database, the main source of information in biomedicine. However, because we were undertaking a citation analysis, we used the Web of Science (WoS) platform to generate a ranking of the top-50 cited articles on cysticercosis and neurocysticercosis, as the WoS includes the MEDLINE database and also provides data on how frequently a particular article has been cited by others.

Two search strategies were combined to identify the included documents: we searched for the medical subject headings (MeSH) term “Cysticercosis” plus natural language appearing in the title and abstract fields. Using a thesaurus enables the precise identification of the literature related to the topic under study, while free-text terms ensure an exhaustive search that includes the most recent papers, which have not yet been assigned descriptors.

The search strategies used were as follows:

1. Cysticercosis (MeSH Heading)
2. Cysticercosis (Title/Abstract) OR Cysticercoses (Title/Abstract) Infection*” (Title/Abstract) OR Coenurosis (Title/Abstract) OR Coenuroses (Title/Abstract) OR “Coenurus cerebralis Infection* (Title/Abstract) OR “Cysticercus cellulosae Infection*” (Title/Abstract) OR “Taenia solium Cysticercosis” (Title/Abstract) OR “Taenia solium Cysticercoses” (Title/Abstract)
3. Neurocysticercosis (Title/Abstract) OR Neurocysticercoses (Title/Abstract) OR Neurocoenurosis (Title/Abstract) OR Neurocoenuroses (Title/Abstract) OR “Central Nervous System Cysticercosis” (Title/Abstract) OR “CNS Cysticercosis” (Title/Abstract) OR “CNS Cysticercoses” (Title/Abstract) OR “Brain Cysticercosis” (Title/Abstract) “Cerebral Coenurosis” (Title/Abstract) OR “Cerebral Coenuroses” (Title/Abstract) OR “Cerebral Cysticercosis” (Title/Abstract) OR “Cerebral Cysticercoses” (Title/Abstract)

In the MeSH thesaurus, the term “Neurocysticercosis” is a specific descriptor for cysticercosis, so all documents indexed with this descriptor were automatically retrieved in the wider search; moreover, both terms (along with their synonyms and variants) were included in the natural language searches. All the search results were combined with the OR operator, searched for in the WoS-Core Collection (WoS-CC) databases, and ranked by the number of citations received to identify the top-50 most cited research papers. All searches were performed in October 2022.

We excluded papers in which cysticercosis or neurocysticercosis was one of several diseases covered (e.g. epidemiological studies on neglected tropical diseases in general or several in particular), as well as generic documents mentioning cysticercosis or neurocysticercosis in other contexts, for example, with regard to diagnostic procedures or clinical studies on other diseases (See **Supplementary table 1,** http://links.lww.com/MD/L698 for the list of excluded papers, with reasons).

We present the top-cited original articles (n = 29) separately from the top-cited reviews/ guidelines (n = 21) due to the different orientation and purpose of these document types. For each document, we recorded the following data: the full reference (authors’ names, journal title, publication year), journal category in the WoS, countries of the first and last authors, and type of document (article, review, meta-analysis, trial). We assessed the full-text article to characterize the topic(s) covered and analyze their content. The citation density was calculated by dividing the number of citations by the journal position in its category in the *Journal Citation Reports*.

Ethical review, because this was an analysis of scientific production and had no human subjects, no ethics review was required. We performed an analysis on routine administrative data (MEDLINE and WoS); consent for publication is not applicable.

## 3. Results

The MEDLINE searches yielded 7860 documents, although only 5411 documents (68.8%) also included in WoS-CC were considered for the analysis. Nineteen documents among the most highly cited were excluded due to their lack of specific focus on cysticercosis or neurocysticercosis.

Table 1 lists the full references and citation data for the top-cited articles (n = 29) and Table 2, the top-cited reviews/guideline (n = 21) on cysticercosis and neurocysticercosis. Of the 21 reviews, 14 (66.7%) were narrative reviews; 4 (19.0%) guidelines, 2 (9.5%), systematic reviews; and 1 (4.5%) each, a meta-analysis.

**Table 1:** Ranking and characteristics of the top-cited original studies on cysticercosis and neurocysticercosis.

Overall rank	Article rank	Publication	Topic	Country of origin		N citations	Citation density
				1^st^ author	Last author		
1	1	Tsang VC, Brand JA, Boyer AE. An enzyme-linked immunoelectrotransfer blot assay and glycoprotein antigens for diagnosing human cysticercosis (Taenia solium). J Infect Dis. 1989;159(1):50-59. doi:10.1093/infdis/159.1.50	Diagnosis, laboratory parameters	USA	USA	638	20.58
4	2	Sotelo J, Guerrero V, Rubio F. Neurocysticercosis: a new classification based on active and inactive forms. A study of 753 cases. Arch Intern Med. 1985;145(3):442-445.	Clinical presentation	Mexico	Mexico	367	10.49
5	3	Osborn AG, Preece MT. Intracranial cysts: radiologic-pathologic correlation and imaging approach. Radiology. 2006;239(3):650-664. doi:10.1148/radiol.2393050823	Imaging	USA	USA	346	24.71
7	4	Schantz PM, Moore AC, Muñoz JL, et al Neurocysticercosis in an Orthodox Jewish community in New York City. N Engl J Med. 1992;327(10):692-695. doi:10.1056/NEJM199209033271004	Clinical presentation	USA	USA	308	11
13	5	Garcia HH, Pretell EJ, Gilman RH, et al A trial of antiparasitic treatment to reduce the rate of seizures due to cerebral cysticercosis. N Engl J Med. 2004;350(3):249-258. doi:10.1056/NEJMoa031294	Therapeutics	Peru	Peru	255	15.94
14	6	McCormick GF, Zee CS, Heiden J. Cysticercosis cerebri. Review of 127 cases. Arch Neurol. 1982;39(9):534-539. doi:10.1001/archneur.1982.00510210004002	Clinical presentation	USA	USA	248	6.53
15	7	Sotelo J, Escobedo F, Rodriguez-Carbajal J, Torres B, Rubio-Donnadieu F. Therapy of parenchymal brain cysticercosis with praziquantel. N Engl J Med. 1984;310(16):1001-1007. doi:10.1056/NEJM198404193101601	Therapeutics	Mexico	Mexico	246	6.83
16	8	Johnson KS, Harrison GB, Lightowlers MW, et al Vaccination against ovine cysticercosis using a defined recombinant antigen. Nature. 1989;338(6216):585-587. doi:10.1038/338585a0	Therapeutics	Australia	Australia	240	24
17	9	Del Brutto OH, Santibañez R, Noboa CA, Aguirre R, Díaz E, Alarcón TA. Epilepsy due to neurocysticercosis: analysis of 203 patients. Neurology. 1992;42(2):389-392. doi:10.1212/wnl.42.2.389	Central nervous system & epilepsy	Peru	Peru	239	8.54
18	10	Wilson M, Bryan RT, Fried JA, et al Clinical evaluation of the cysticercosis enzyme-linked immunoelectrotransfer blot in patients with neurocysticercosis. J Infect Dis. 1991;164(5):1007-1009. doi:10.1093/infdis/164.5.1007	Diagnosis, laboratory parameters	USA	USA	228	7.76
21	11	Bowles J, McManus DP. Genetic characterization of the Asian Taenia, a newly described taeniid cestode of humans. Am J Trop Med Hyg. 1994;50(1):33-44	Parasitology	Australia	Australia	206	7.92
22	12	Dorny P, Phiri IK, Vercruysse J, et al A Bayesian approach for estimating values for prevalence and diagnostic test characteristics of porcine cysticercosis. Int J Parasitol. 2004;34(5):569-576. doi:10.1016/j.ijpara.2003.11.014	Epidemiology	Belgium	Belgium	200	12.5
24	13	Medina MT, Rosas E, Rubio-Donnadieu F, Sotelo J. Neurocysticercosis as the main cause of late-onset epilepsy in Mexico. Arch Intern Med. 1990;150(2):325-327	Central nervous system & epilepsy	Mexico	Mexico	195	6.5
27	14	Garcia HH, Gilman R, Martinez M, et al Cysticercosis as a major cause of epilepsy in Peru. The Cysticercosis Working Group in Peru (CWG). Lancet. 1993;341(8839):197-200. doi:10.1016/0140-6736(93)90064-n	Central nervous system & epilepsy	Peru	Peru	174	6.44
28	15	Yamasaki H, Allan JC, Sato MO, et al DNA differential diagnosis of teniasis and cysticercosis by multiplex PCR. J Clin Microbiol. 2004;42(2):548-553. doi:10.1128/JCM.42.2.548-553.2004	Diagnosis, laboratory parameters	Japan	Japan	168	10.5
29	16	Sotelo J, del Brutto OH, Penagos P, et al Comparison of therapeutic regimen of anticysticercal drugs for parenchymal brain cysticercosis. J Neurol. 1990;237(2):69-72. doi:10.1007/BF00314663	Therapeutics	Mexico	Mexico	164	5.47
31	17	Vazquez V, Sotelo J. The course of seizures after treatment for cerebral cysticercosis. N Engl J Med. 1992;327(10):696-701. doi:10.1056/NEJM199209033271005	Central nervous system & epilepsy	Mexico	Mexico	161	6.71
33	18	Sarti E, Schantz PM, Plancarte A, et al Prevalence and risk factors for *Taenia solium* teniasis and cysticercosis in humans and pigs in a village in Morelos, Mexico. Am J Trop Med Hyg. 1992;46(6):677-685. doi:10.4269/ajtmh.1992.46.677	Epidemiology	USA	Mexico	160	10.67
34	19	Montano SM, Villaran MV, Ylquimiche L, et al Neurocysticercosis: association between seizures, serology, and brain CT in rural Peru. Neurology. 2005;65(2):229-233. doi:10.1212/01.wnl.0000168828.83461.09	Central nervous system & epilepsy	Peru	Peru	160	10
37	20	Takayanagui OM, Jardim E. Therapy for neurocysticercosis. Comparison between albendazole and praziquantel. Arch Neurol. 1992;49(3):290-294. doi:10.1001/archneur.1992.00530270106026	Therapeutics	Brazil	Brazil	153	5.46
39	21	Escobedo F, Penagos P, Rodriguez J, Sotelo J. Albendazole therapy for neurocysticercosis. Arch Intern Med. 1987;147(4):738-741.	Therapeutics	Mexico	Mexico	146	48.67
41	22	Brandt JR, Geerts S, De Deken R, et al A monoclonal antibody-based ELISA for the detection of circulating excretory-secretory antigens in Taenia saginata cysticercosis. Int J Parasitol. 1992;22(4):471-477. doi:10.1016/0020-7519(92)90148-e.	Diagnosis, laboratory parameters	Belgium	Belgium	145	5.18
42	23	Terrazas LI, Bojalil R, Govezensky T, Larralde C. Shift from an early protective Th1-type immune response to a late permissive Th2-type response in murine cysticercosis (Taenia crassiceps). J Parasitol. 1998;84(1):74-81.	Immunology	Mexico	Mexico	144	8.47
43	24	Phiri IK, Ngowi H, Afonso S, et al The emergence of *Taenia solium* cysticercosis in Eastern and Southern Africa as a serious agricultural problem and public health risk. Acta Trop. 2003;87(1):13-23. doi:10.1016/s0001-706 × (03)00051-2.	Epidemiology	Zambia	Denmark	144	6.55
44	25	Rosas N, Sotelo J, Nieto D. ELISA in the diagnosis of neurocysticercosis. Arch Neurol. 1986;43(4):353-356. doi:10.1001/archneur.1986.00520040039016	Diagnosis, laboratory parameters	Mexico	Mexico	143	17.87
45	26	Bern C, Garcia HH, Evans C, et al Magnitude of the disease burden from neurocysticercosis in a developing country. Clin Infect Dis. 1999;29(5):1203-1209. doi:10.1086/313470	Epidemiology	USA	USA	142	6.76
46	27	Gonzalez AE, Cama V, Gilman RH, et al Prevalence and comparison of serologic assays, necropsy, and tongue examination for the diagnosis of porcine cysticercosis in Peru. Am J Trop Med Hyg. 1990;43(2):194-199. doi:10.4269/ajtmh.1990.43.194	Epidemiology	Peru	Peru	142	6.76
48	28	Shandera WX, White AC Jr, Chen JC, Diaz P, Armstrong R. Neurocysticercosis in Houston, Texas. A report of 112 cases. Medicine (Baltimore). 1994;73(1):37-52. doi:10.1097/00005792-199401000-00004	Clinical presentation	USA	USA	139	5.35
50	29	Sotelo J, Escobedo F, Penagos P. Albendazole vs praziquantel for therapy for neurocysticercosis. A controlled trial. Arch Neurol. 1988;45(5):532-534. doi:10.1001/archneur.1988.00520290064015	Therapeutics	Mexico	Mexico	137	4.28

**Table 2:** Ranking and characteristics of the top-cited reviews on cysticercosis and neurocysticercosis.

Overall rank	Review rank	Publication	Topic	Country of origin		N citations	Citation density
				1^st^ author	Last author		
2	1	Del Brutto OH, Rajshekhar V, White AC Jr, et al Proposed diagnostic criteria for neurocysticercosis. Neurology. 2001;57(2):177-183. doi:10.1212/wnl.57.2.177	Diagnosis, clinical presentation	Peru	Peru	537	28.29
3	2	García HH, Gonzalez AE, Evans CA, Gilman RH; Cysticercosis Working Group in Peru. *Taenia solium* cysticercosis. Lancet. 2003;362(9383):547-556. doi:10.1016/S0140-6736(03)14117-7	All aspects	Peru	USA	456	26.82
6	3	Garcia HH, Del Brutto OH; Cysticercosis Working Group in Peru. Neurocysticercosis: updated concepts about an old disease. Lancet Neurol. 2005;4(10):653-661. doi:10.1016/S1474-4422(05)70194-0	All aspects	Peru	Peru	318	21.2
8	4	García HH, Evans CA, Nash TE, et al Current consensus guidelines for treatment of neurocysticercosis. Clin Microbiol Rev. 2002;15(4):747-756. doi:10.1128/CMR.15.4.747-756.2002	Therapeutics	Peru	USA	278	15.44
9	5	Del Brutto OH, Sotelo J. Neurocysticercosis: an update. Rev Infect Dis. 1988;10(6):1075-1087. doi:10.1093/clinids/10.6.1075	All aspects	Peru	Mexico	263	8.22
10	6	Garcia HH, Nash TE, Del Brutto OH. Clinical symptoms, diagnosis, and treatment of neurocysticercosis. Lancet Neurol. 2014;13(12):1202-1215. doi:10.1016/S1474-4422(14)70094-8	All aspects	Peru	Peru	262	43.67
7	11	Ndimubanzi PC, Carabin H, Budke CM, et al A systematic review of the frequency of neurocyticercosis with a focus on people with epilepsy. PLoS Negl Trop Dis. 2010;4(11):e870. Published 2010 Nov 2. doi:10.1371/journal.pntd.0000870	Central nervous system & epilepsy	USA	USA	258	25.8
12	8	White AC Jr. Neurocysticercosis: a major cause of neurological disease worldwide. Clin Infect Dis. 1997;24(2):101-115. doi:10.1093/clinids/24.2.101	Central nervous system & epilepsy	USA	USA	256	11.13
19	9	White AC Jr. Neurocysticercosis: updates on epidemiology, pathogenesis, diagnosis, and management. Annu Rev Med. 2000;51:187-206. doi:10.1146/annurev.med.51.1.187	All aspects	USA	USA	221	11.05
20	10	Carpio A. Neurocysticercosis: an update. Lancet Infect Dis. 2002;2(12):751-762. doi:10.1016/s1473-3099(02)00454-1	All aspects	Ecuador	Ecuador	207	11.5
23	11	Carpio A, Escobar A, Hauser WA. Cysticercosis and epilepsy: a critical review. Epilepsia. 1998;39(10):1025-1040. doi:10.1111/j.1528-1157.1998.tb01287.x	Central nervous system & epilepsy	Ecuador	USA	196	5.16
25	12	Carabin H, Ndimubanzi PC, Budke CM, et al Clinical manifestations associated with neurocysticercosis: a systematic review. PLoS Negl Trop Dis. 2011;5(5):e1152. doi:10.1371/journal.pntd.0001152	Clinical	USA	USA	190	21.11
26	13	Román G, Sotelo J, Del Brutto O, et al A proposal to declare neurocysticercosis an international reportable disease. Bull World Health Organ. 2000;78(3):399-406	Epidemiology	USA	Brazil	188	9.4
30	14	Sciutto E, Fragoso G, Fleury A, et al *Taenia solium* disease in humans and pigs: an ancient parasitosis disease rooted in developing countries and emerging as a major health problem of global dimensions. Microbes Infect. 2000;2(15):1875-1890. doi:10.1016/s1286-4579(00)01336-8	All aspects	Mexico	Mexico	162	8.1
32	15	Del Brutto OH, Wadia NH, Dumas M, Cruz M, Tsang VC, Schantz PM. Proposal of diagnostic criteria for human cysticercosis and neurocysticercosis. J Neurol Sci. 1996;142(1-2):1-6. doi:10.1016/0022-510 × (96)00130-x	Diagnosis, clinical presentation	Peru	USA	161	5.75
35	16	Rickard MD, Williams JF. Hydatidosis/cysticercosis: immune mechanisms and immunization against infection. Adv Parasitol. 1982;21:229-296. doi:10.1016/s0065-308 × (08)60277-8	Imaging	Australia	USA	160	5.71
36	17	Nash TE, Del Brutto OH, Butman JA, et al Calcific neurocysticercosis and epileptogenesis. Neurology. 2004;62(11):1934-1938. doi:10.1212/01.wnl.0000129481.12067.06	Central nervous system & epilepsy	USA	Peru	160	4.21
38	18	García HH, Del Brutto OH. Imaging findings in neurocysticercosis. Acta Trop. 2003;87(1):71-78. doi:10.1016/s0001-706 × (03)00057-3	Imaging	Peru	Peru	147	8.65
40	19	Del Brutto OH, Nash TE, White AC Jr, et al Revised diagnostic criteria for neurocysticercosis. J Neurol Sci. 2017;372:202-210. doi:10.1016/j.jns.2016.11.045	Diagnosis, clinical presentation	Peru	Peru	146	4.42
47	20	Del Brutto OH, Roos KL, Coffey CS, García HH. Meta-analysis: Cysticidal drugs for neurocysticercosis: albendazole and praziquantel. Ann Intern Med. 2006;145(1):43-51. doi:10.7326/0003-4819-145-1-200607040-00009	Therapeutics	Peru	Peru	141	4.7
49	21	Wallin MT, Kurtzke JF. Neurocysticercosis in the United States: review of an important emerging infection. Neurology. 2004;63(9):1559-1564. doi:10.1212/01.wnl.0000142979.98182.ff	Epidemiology	USA	USA	138	8.63

The 50 studies were published in 27 journals (Table 3), but 6 journals contained 44% of the included articles. *Neurology* was the most prolific, publishing 10% of top-cited papers, followed by the *New England Journal of Medicine, Archives of Neurology, Clinical Infectious Diseases, Archives of Internal Medicine* and the *American Journal of Tropical Medicine and Hygiene*. Regarding the dominant specialties, these comprised Neurology (30% of documents), General & Internal Medicine (28%) and Parasitology (22%). Other disciplines showed smaller contributions (Infectious Diseases, 14%; Microbiology, 6%; and Radiology, 2%) to the set of high-impact studies on cysticercosis and neurocysticercosis (Table 3).

**Table 3:** Distribution of the top-50 cited papers on cysticercosis and neurocysticercosis by journal and category.

Journal	All (N = 50)		Articles (N = 29)		Reviews (N = 21)	
	N docs	%	N docs	%	N docs	%
Neurology	5	10.0	2	6.9	3	14.3
New England Journal of Medicine	4	8.0	4	13.8	0	0.0
Archives of Neurology	4	8.0	4	13.8	0	0.0
Clinical Infectious Diseases	3	6.0	1	3.4	2	9.5
Archives of Internal Medicine	3	6.0	3	10.3	0	0.0
American Journal of Tropical Medicine and Hygiene	3	6.0	3	10.3	0	0.0
Journal of Infectious Diseases	2	4.0	2	6.9	0	0.0
Lancet	2	4.0	1	3.4	1	4,8
Journal of the Neurological Sciences	2	4.0	0	0.0	2	4.8
Lancet Neurology	2	4.0	0	0.0	2	9.5
Acta Tropica	2	4.0	1	3.4	1	4.8
International Journal for Parasitology	2	4.0	2	6.9	0	0.0
PLOS Neglected Tropical Diseases	2	4.0	0	0.0	2	9.5
Lancet Infectious Diseases	1	2.0	0	0.0	1	4.8
Annals of Internal Medicine	1	2.0	0	0.0	1	4.8
Annual Review of Medicine	1	2.0	0	0.0	1	4.8
Bulletin of the World Health Organization	1	2.0	0	3.4	1	4.8
Medicine	1	2.0	1	3.4	0	0.0
Nature	1	2.0	1	3.4	0	0.0
Clinical Microbiology Reviews	1	2.0	0	0.0	1	4.8
Journal of Clinical Microbiology	1	2.0	1	3.4	0	0.0
Microbes and Infection	1	2.0	0	0.0	1	4.8
Epilepsia	1	2.0	0	0.0	1	4.8
Journal of Neurology	1	2.0	1	3.4	0	0.0
Advances in Parasitology	1	2.0	0	0.0	1	4.8
Journal of Parasitology	1	2.0	1	3.4	0	0.0
Radiology	1	2.0	1	3.4	0	0.0
Category						
Neurology	15	30	7	24.1	8	38.1
General & Internal Medicine	14	28	10	34.5	4	19.0
Parasitology	11	22	7	24.1	4	19.0
Infectious Diseases	7	14	4	13.8	3	14.3
Microbiology	3	6	1	3.4	2	9.5
Radiology	1	2	1	3.4	0	0.0

The most frequent countries of origin among first authors were the USA and Peru (30% each), followed by Mexico (20%). Last authors were most often from the USA (34%), followed by Mexico and Peru (24% each), as shown in Table 4. The authors appearing most frequently in these leadership roles (first and last position) were García HH, Del-Brutto, OH and Sotelo J (Table 5).

**Table 4:** Distribution of the top-50 cited papers on cysticercosis and neurocysticercosis, by country of origin of first and last authors.

	All (N = 50)		Articles (N = 29)		Reviews (N = 21)	
	N docs	%	N docs	%	N docs	%
Country of origin, 1^st^ author						
USA	15	30.0	8	27.6	7	33.3
Peru	15	30.0	5	17.2	10	47.6
Mexico	10	20.0	9	31.0	1	4.8
Australia	3	6.0	2	6.9	1	4.8
Belgium	2	4.0	0	0.0	2	9.5
Brazil	1	2.0	1	2.4	0	0.0
Japan	1	2.0	1	3.4	0	0.0
Zambia	1	2.0	1	3.4	0	0.0
Country of origin, last author						
USA	17	34.0	7	24.1	10	47.6
Peru	12	24.0	5	17.6	7	33.3
Mexico	12	24.0	10	34.5	2	9.5
Australia	2	4.0	2	6.9	0	0.0
Belgium	2	4.0	2	6.9	0	0.0
Brazil	2	4.0	1	3.4	1	4.8
Japan	1	2.0	1	3.4	0	0.0
Ecuador	1	2.0	0	0.0	1	4.8

**Table 5:** First and last authors with more than one of the top-50 cited papers on cysticercosis and neurocysticercosis, by number and type of publications.

	All (N = 50)		Articles (N = 29)		Reviews (N = 21)	
	N docs	%	N docs	N docs	%	N docs
1^st^ author						
García HH	7	14.0	2	6.9	5	23.8
Del-Brutto OH	6	12.0	1	3.4	5	23.8
Sotelo J	4	8.0	4	13.8	0	0.0
Carpio A	2	4.0	0	0.0	2	9.5
Last author						
García HH	5	10.0	1	3.4	4	10.0
Sotelo J	4	4.0	3	10.3	1	4.8
Del-Brutto OH	3	6.0	0	0.0	3	14.3
Gilman RH	3	6.0	1	3.4	2	9.5
Rubio F	3	6.0	3	10.3	0	0.0
Flisser A	2	4.0	2	6.9	0	0.0
Larralde C	2	4.0	1	3.4	1	4.8
White AC	2	4.0	0	0.0	2	9.5

Most (80%) of the top-50 cited documents were published from 1990 on (72% of articles and 90.5% of reviews) (Table 6). The most frequent topics in the included documents were the central nervous system & epilepsy and therapeutics (18% each, Table 7). Other prominent topics were epidemiology and diagnostics.

**Table 6:** Top-50 cited papers on cysticercosis and neurocysticercosis, by decade of publication.

Period	All (N = 50)		Articles (N = 29)		Reviews (N = 21)	
	N docs	%	N docs	N docs	%	N docs
1982–1989	10	20.0	8	27.0	2	9.5
1990–1999	18	36.0	15	51.7	3	14.3
2000–2017	22	44.0	6	20.7	16	76.2

**Table 7:** Top-50 cited papers on cysticercosis and neurocysticercosis, by topic.

Topics	All (N = 50)		Articles (N = 29)		Reviews (N = 21)	
	N docs	%	N docs	%	N docs	%
Central nervous system & epilepsy	9	18	5	17.2	4	19
Therapeutics	9	18	7	24.1	2	9.5
All aspects	7	14	0	0	7	33.3
Epidemiology	7	14	5	17.2	2	9.5
Clinical presentation	5	10	4	13.8	1	4.8
Diagnosis, laboratory parameters	5	10	5	17.2	0	0
Imaging	3	6	1	3.4	2	9.5
Diagnosis, clinic	3	6	0	0	3	14.3
Parasitology	1	2	1	3.4	0	0
Immunology	1	2	1	3.4	0	0

## 4. Discussion

Several methodologies exist for measuring research impact, but the most commonly used to identify influential works are still the number of citations and the journal citation rank lists,^[8]^ as shown by a myriad of studies in areas including parasitology, tropical medicine, and infectious diseases, among others.^[9–14]^ An analysis of the top-cited articles illustrates how knowledge accumulates over time, so this study aimed to determine which articles on cysticercosis and neurocysticercosis have been most influential in the field.

In that regard, the evolution of scientific research on cysticercosis and neurocysticercosis has been driven by efforts to improve central nervous system problems (e.g. epilepsy, a complication of neurocysticercosis), treatment-related aspects (by means of 4 clinical trials), diagnostics, and epidemiological knowledge. The most cited article (638 citations) described an enzyme-linked immunoelectrotransfer blot-based assay for diagnosing human cysticercosis, published by Tsang et al^[15]^ The most frequently cited review (537 citations) was led by Del-Brutto^[16]^ and covered diagnostic criteria for neurocysticercosis. Other papers in the top 5 included a narrative review, led by García et al^[17]^ about *Taenia solium* cysticercosis and its impact in neurological disease; a study by Sotelo et al^[18]^ proposing diagnostic criteria for neurocysticercosis, which were the basis for the updated diagnosis criteria for neurocysticercosis proposed in Del-Brutto et al^[16]^ review; and an original article by Osborn & Preece,^[19]^ focusing on the differential diagnosis of cysticercosis and neurocysticercosis based on the study of many different intracranial cysts.

Other prominent contributions related to diagnostics included the paper by Schantz et al,^[20]^ who studied 4 patients with recurrent seizures and brain lesions that were radiologically consistent with the presence of cysticercus; the authors concluded that recently arrived family members from countries where *T solium* was endemic were the most likely sources of infection. Additionally, Wilson et al^[21]^ described the serological diagnosis of neurocysticercosis by enzyme-linked immunoelectrotransfer blot, while Yamasaki et al^[22]^ described the molecular diagnosis of cysticercosis (DNA differential diagnosis of teniasis and cysticercosis by multiplex PCR). Del-Brutto et al^[23]^ proposed diagnostic criteria for human cysticercosis and neurocysticercosis; Montano et al^[24]^ analyzed the association between seizures, serology, and brain computed tomography (CT) abnormalities compatible with neurocysticercosis in Peru; and Rosas et al^[25]^ studied enzyme-linked immunosorbent assay for diagnosing neurocysticercosis.

With regard to high-impact papers on treatment, García et al^[26]^ reported a double-blind, placebo-controlled trial of albendazole plus dexamethasone versus placebo for neurocysticercosis; McCormick et al^[27]^ analyzed 127 cases of cysticercosis cerebri or neurocysticercosis in USA; and Sotelo et al^[28]^ described a series of 37 cases of neurocysticercosis treated with praziquantel. Six years later, the same group (Sotelo et al)^[29]^ published a 4-arm randomized controlled trial of neurocysticercosis treatments (praziquantel 50 mg/kg/day for 15 days versus praziquantel 50 mg/kg/day for 8 days versus albendazole 15 mg/kg/day for 30 days versus albendazole 15 mg/kg/day for 8 days); at 3 months’ follow-up, both praziquantel and albendazole were shown to be effective in eliminating cystic lesions on CT scans. In addition, Takayanagui & Jardim^[30]^ published a clinical trial of albendazole and praziquantel; Escobedo et al^[31]^ described treatment with albendazole; Del Brutto et al^[32]^ performed a meta-analysis of cysticidal drugs for neurocysticercosis; and Sotelo et al^[33]^ reported a controlled trial of albendazole versus praziquantel (20 patients in each group) for neurocysticercosis.

Among the papers that focused on epilepsy, Del-Brutto et al^[34]^ described 203 patients with epilepsy secondary to neurocysticercosis; Medina et al^[35]^ analyzed 100 Mexican patients with epilepsy that started after the age of 25 years—half the cases were due to neurocysticercosis; and García et al^[36]^ analyzed the relationship between cysticercosis and epilepsy in Peru, finding that 12% of patients with epilepsy had positive serology for cysticercosis.

Other outstanding contributions among the 50 most cited papers include a proposal for vaccination against ovine cysticercosis in sheep^[37]^; an article about a genetic characterization of the Asian Taenia^[38]^; a Bayesian approach for estimating prevalence and diagnostic test characteristics of porcine cysticercosis^[39]^; a proposal to declare neurocysticercosis an international reportable disease, published in the *Bulletin of the World Health Organization*^[40]^; an analysis of the disease burden due to neurocysticercosis in a developing country^[41]^; an analysis of prevalence and risk factors for *T solium* in Mexico^[20]^; development of a monoclonal antibodies-based capture enzyme-linked immunosorbent assay of circulating parasite antigens *of T solium*^[42]^; a research paper about the immune response in murine cysticercosis^[43]^; and an epidemiological report of the emergence of *T solium* cysticercosis in Eastern and Southern Africa.^[44]^

Most (80%) of the top-50 cited studies were published in 1990 or later (72% of articles and 90.5% of reviews), confirming the increase in research interest around the end of the century. This result is also associated with the phenomenon of obsolescence, and the likelihood that researchers will have a greater interest in more recent studies.^[45]^ Of note, and in contrast to the typical situation in other areas of knowledge—characterized by the hegemony of authors from the USA or Europe among the most cited and seminal contributions^[46]^ is the prominent position of authors from low- and middle-income countries. Another relevant finding is the high number of reviews among the top-cited documents (42%), well above the proportion of reviews relative to all the documents in the field (11.1%). This concentration of citations in reviews is a well-known phenomenon, but it highlights the relevance of this type of study for advancing knowledge.^[47]^ That said, the performance of systematic reviews or other studies producing high-level scientific evidence should be encouraged to a greater extent, as their presence among the top-50 cited papers is negligible compared to the narrative reviews included in this set of documents.^[48]^

This study has several limitations. First of all, citation analyses provide important information about a scientific paper impact, visibility, and use of the information contained therein, but they do not represent a measure of methodological quality or scientific evidence. Moreover, since the analysis focused on papers published in journals with an impact factor in WoS-CC, we may have missed relevant articles from non-English-speaking and endemic countries.^[49,50]^ Other possible biases should also be considered, such as the tendency of some authors to omit citations of studies reporting well-known findings, or the association between the year of publication and the accumulation of citations, which may have led to overlooking important but recent papers that have not^[50]^ yet garnered sufficient citations to be ranked within the top 50. Despite these limitations, citation analyses of top-cited documents are widely used to rank and evaluate a research field, and they provide information of great value to researchers and professionals in the area.

## 5. Conclusions

Our study provides information related to the dissemination of knowledge in recent decades about cysticercosis and neurocysticercosis, which may be helpful to researchers and professionals seeking to understand the research areas generating the most impact. Our results show the relevance of neurology and general and internal medicine in the area; the research leadership of Peru and Mexico, together with the USA; and the keen research interest in the central nervous system & epilepsy, diagnostics, and therapeutic approaches in relation to cysticercosis and neurocysticercosis. We also identify the most cited articles and reviews of reference in relation to all these aspects.

## Author contributions

**Conceptualization:** Gregorio Gonzalez-Alcaide, Nestor Sosa, Jose-Manuel Ramos-Rincon.

**Data curation:** Fatima Valero-Samper, Jose-Manuel Ramos-Rincon.

**Formal analysis:** Gregorio Gonzalez-Alcaide, Jose-Manuel Ramos-Rincon.

**Funding acquisition:** Jose-Manuel Ramos-Rincon.

**Investigation:** Nestor Sosa, Fatima Valero-Samper, Isabel Belinchon-Romero.

**Methodology:** Gregorio Gonzalez-Alcaide.

**Project administration:** Jose-Manuel Ramos-Rincon.

**Writing – original draft:** Gregorio Gonzalez-Alcaide, Fatima Valero-Samper, Isabel Belinchon-Romero, Jose-Manuel Ramos-Rincon.

**Writing – review & editing:** Gregorio Gonzalez-Alcaide, Nestor Sosa, Isabel Belinchon-Romero, Jose-Manuel Ramos-Rincon.

## Supplementary Material


