# Supplementary material for: Top-50 cited articles on cysticercosis and neurocysticercosis
Source: Medicine (Baltimore). 2024 Mar 1;103(9):e37268. doi: 10.1097/MD.0000000000037268 (PMC10906643; doi:10.1097/MD.0000000000037268)
Supplement: Supplementary file 1 [file medi-103-e37268-s001.docx]

**Supplementary table 1. List of excluded papers, with reasons).**

| Rank | Publication | Type of article | Reason of exclusion | Citations  *(n)* | Citation  Density |
| --- | --- | --- | --- | --- | --- |
| 1 | Chai JY, Darwin Murrell K, Lymbery AJ. Fish-borne parasitic zoonoses: status and issues. *Int J Parasitol*. 2005;35(11-12):1233-1254. https://doi.org/10.1016/j.ijpara.2005.07.013 | Review | The article is about meat-borne zoonoses such as trichinellosis and cysticercosis, and fish-borne parasitic zoonoses, caused by trematodes, cestodes and nematodes | 530 | 35,33 |
| 2 | Hotez, P. J., Bottazzi, M. E., Franco-Paredes, C., Ault, S. K., & Periago, M. R. (2008). The neglected tropical diseases of Latin America and the Caribbean: a review of disease burden and distribution and a roadmap for control and elimination. *PLoS neglected tropical diseases*, *2*(9), e300. https://doi.org/10.1371/journal.pntd.0000300 | Review | The article is about tropical diseases that are the most common infections of the poorest people living in the Latin American and Caribbean region, such as helminth infections and Chagas disease. | 455 | 37,91 |
| 3 | Torgerson, P. R., Devleesschauwer, B., Praet, N., Speybroeck, N., Willingham, A. L., Kasuga, F., Rokni, M. B., Zhou, X. N., Fèvre, E. M., Sripa, B., Gargouri, N., Fürst, T., Budke, C. M., Carabin, H., Kirk, M. D., Angulo, F. J., Havelaar, A., & de Silva, N. (2015). World Health Organization Estimates of the Global and Regional Disease Burden of 11 Foodborne Parasitic Diseases, 2010: A Data Synthesis. *PLoS medicine*, *12*(12), e1001920. https://doi.org/10.1371/journal.pmed.1001920 | Article | The article is about estimating the global and regional human disease burden of helminth diseases and toxoplasmosis that may be attributed to contaminated food. | 330 | 66 |
| 4 | Preux, P. M., & Druet-Cabanac, M. (2005). Epidemiology and aetiology of epilepsy in sub-Saharan Africa. *The Lancet. Neurology*, *4*(1), 21–31. https://doi.org/10.1016/S1474-4422(04)00963-9 | Review | The article is about the incidence, prognosis and etiology for epilepsy in sub-Saharan Africa. | 305 | 20,33 |
| 5 | Misra, U. K., & Kalita, J. (2010). Overview: Japanese encephalitis. *Progress in neurobiology*, *91*(2), 108–120. https://doi.org/10.1016/j.pneurobio.2010.01.008 | Review | The article is about the incidence, symptoms and diagnosis of Japanese encephalitis, and about coinfection of JE and cysticercosis. | 240 | 7,74 |
| 6 | Kwee, R. M., & Kwee, T. C. (2007). Virchow-Robin spaces at MR imaging. *Radiographics: a review publication of the Radiological Society of North America, Inc*, *27*(4), 1071–1086. https://doi.org/10.1148/rg.274065722 | Article | The article is about the locations of Virchow-Robin (VR) spaces and the signal intensity characteristics and locations to differentiate various pathologic conditions. | 233 | 17,92 |
| 7 | Hotez P. J. (2008). Neglected infections of poverty in the United States of America. *PLoS neglected tropical diseases*, *2*(6), e256. https://doi.org/10.1371/journal.pntd.0000256 | Review | The article is about neglected infections of poverty in USA, such as helminth infections, toxocariasis, strongyloidiasis, ascariasis, and cysticercosis. | 222 | 18,5 |
| 8 | Lustigman, S., Prichard, R. K., Gazzinelli, A., Grant, W. N., Boatin, B. A., McCarthy, J. S., & Basáñez, M. G. (2012). A research agenda for helminth diseases of humans: the problem of helminthiases. *PLoS neglected tropical diseases*, *6*(4), e1582. https://doi.org/10.1371/journal.pntd.0001582 | Review | The article is about helminthiases in human populations, including onchocerciasis, lymphatic filariasis, soil-transmitted helminthiases, schistosomiasis and taeniasis/cysticercosis. | 208 | 26 |
| 9 | Torgerson, P. R., & Macpherson, C. N. (2011). The socioeconomic burden of parasitic zoonoses: global trends. *Veterinary parasitology*, *182*(1), 79–95. https://doi.org/10.1016/j.vetpar.2011.07.017 | Article | The article is about zoonotic transmission of parasites, such as toxoplasmosis, food borne trematode infections, cysticercosis, echinococcosis, leishmaniosis. | 207 | 23 |
| 10 | Chung, J. K., Kim, Y. K., Kim, S. K., Lee, Y. J., Paek, S., Yeo, J. S., Jeong, J. M., Lee, D. S., Jung, H. W., & Lee, M. C. (2002). Usefulness of 11C-methionine PET in the evaluation of brain lesions that are hypo- or isometabolic on 18F-FDG PET. *European journal of nuclear medicine and molecular imaging*, *29*(2), 176–182. https://doi.org/10.1007/s00259-001-0690-4 | Article | The article is about the use of 11C-methionine PET to evaluate brain lesions that are not detected on 18F-FDG PET. | 201 | 11,17 |
| 11 | Senanayake, N., & Román, G. C. (1993). Epidemiology of epilepsy in developing countries. *Bulletin of the World Health Organization*, *71*(2), 247–258. | Review | The article is about the epidemiology of epilepsy in developing countries in terms of its incidence, prevalence, seizure type, mortality data, and etiological factors. | 197 | 7,3 |
| 12 | Apuzzo, M. L., Chikovani, O. K., Gott, P. S., Teng, E. L., Zee, C. S., Giannotta, S. L., & Weiss, M. H. (1982). Transcallosal, interfornicial approaches for lesions affecting the third ventricle: surgical considerations and consequences. *Neurosurgery*, *10*(5), 547–554. https://doi.org/10.1227/00006123-198205000-00001 | Article | The article is about a surgical technique using a direct transcallosal interfornicial to treat lesions affecting the 3rd ventricle. | 196 | 8,9 |
| 13 | Moskowitz, L. B., Hensley, G. T., Chan, J. C., Gregorios, J., & Conley, F. K. (1984). The neuropathology of acquired immune deficiency syndrome. *Archives of pathology & laboratory medicine*, *108*(11), 867–872. | Article | This article reviews the neuropathologic characteristics of 52 cases of acquired immune deficiency syndrome at autopsy. | 184 | 5,11 |
| 14 | Graeff-Teixeira, C., da Silva, A. C., & Yoshimura, K. (2009). Update on eosinophilic meningoencephalitis and its clinical relevance. *Clinical microbiology reviews*, *22*(2), 322–348. https://doi.org/10.1128/CMR.00044-08 | Review | This article is about eosinophilic meningoencephalitis, that is caused by a variety of helminthic infections, sucha as angiostrongyliasis, toxocariasis, cysticercosis, schistosomiasis. | 169 | 15,36 |
| 15 | Wu, Z., Mittal, S., Kish, K., Yu, Y., Hu, J., & Haacke, E. M. (2009). Identification of calcification with MRI using susceptibility-weighted imaging: a case study. *Journal of magnetic resonance imaging: JMRI*, *29*(1), 177–182. https://doi.org/10.1002/jmri.21617 | Article | The article is about the use of Susceptibility weighted imaging to identify calcification, such as oligodendroglioma, cysticercosis or physiologic calcifications. | 162 | 14,73 |
| 16 | Craig, P. S., Rogan, M. T., & Allan, J. C. (1996). Detection, screening and community epidemiology of taeniid cestode zoonoses: cystic echinococcosis, alveolar echinococcosis and neurocysticercosis. *Advances in parasitology*, 38, 169–250. https://doi.org/10.1016/s0065-308x(08)60035-4 | Review | This article is about diagnosis and screening of taeniid cestode zoonoses, such as cystic echinococcosis, alveolar echinococcosis and neurocysticercosis. | 154 | 6,42 |
| 17 | Matossian, R. M., Rickard, M. D., & Smyth, J. D. (1977). Hydatidosis: a global problem of increasing importance. Bulletin of the World Health Organization, 55(4), 499–507. | Review | This article is about the occurrence of hydatid disease due to Echinococcus granulosus and E. multilocularis. | 151 | 3,51 |
| 18 | Prichard, R. K., Basáñez, M.-G., Boatin, B. A., McCarthy, J. S., García, H. H., Yang, G.-J., Sripa, B., & Lustigman, S. (2012). A research agenda for helminth diseases of humans: Intervention for control and elimination. *PLoS Neglected Tropical Diseases*, *6*(4). https://doi.org/10.1371/journal.pntd.0001549 | Review | The article is about the epidemiology, diagnosis and treatment of human helminthiases. | 143 | 4,2 |
| 17 | Ellner, J. J., & Bennett, J. E. (1976). Chronic meningitis. Medicine, 55(5), 341–369. https://doi.org/10.1097/00005792-197609000-00001 | Review | The article is about the syndrome of chronic meningitis. | 137 | 3,11 |
